# Exploring the Relationship between Disordered Sleep and Mood in Male Anorexia Nervosa: An Actigraphy Study

**DOI:** 10.3390/nu15092176

**Published:** 2023-05-02

**Authors:** Mengyu Lim, Ruoxin Kou, Gianluca Esposito, Aisha Jawed, Dagmara Dimitriou, Stephen A. Mangar

**Affiliations:** 1 Psychology Program, School of Social Sciences, Nanyang Technological University, 48 Nanyang Avenue, Singapore 639818, Singapore; mengyu001@e.ntu.edu.sg; 2 Sleep Education and Research Laboratory, UCL Institute of Education, London WC1H 0AA, UK; ruoxin.kou.21@ucl.ac.uk (R.K.); aisha.jawed.15@ucl.ac.uk (A.J.); 3 Affiliative Behaviour and Physiology Lab, Department of Psychology and Cognitive Science, University of Trento, 84 Corso Bettini, 38068 Rovereto, Italy; gianluca.esposito@unitn.it; 4 Department of Clinical Oncology, Imperial College Healthcare NHS Trust, Charing Cross Hospital, London W6 8RF, UK

**Keywords:** eating disorder, anorexia nervosa, sleep, actigraphy, male, mood, emotion, depression, anxiety, stress

## Abstract

Eating disorders (EDs), including anorexia nervosa (AN), are severe psychological disorders that affect individuals’ eating behaviours and body perception. Previous research has shown that people with EDs often report poorer sleep. Some literature has suggested that it is mood dysregulation that mediates the link between EDs and sleep. However, the majority of previous studies only focused on females, while male ED patients have been overlooked. Therefore, the present study aimed to investigate the relationships between EDs, mood, and sleep among male ED patients. Using a mixture of actigraphy recordings and self-reported questionnaires, the current study analysed a total 33 adult male participants diagnosed with AN. The participants first wore an actigraphy device for seven continuous days, following which their ED severity and mood were assessed by the Eating Disorder Examination Questionnaire (EDE-Q) and Depression Anxiety Stress Scale (DASS), respectively. The descriptive actigraphy results suggested that, similar to females, males with AN also showed disturbed sleep, including insomnia, sleep fragmentation, low sleep efficiency, and increased napping sessions. However, when ED severity was correlated against actigraphy data and mood, no significant relationships were found between them. Thus, it was suggested that future studies may investigate discrete ED symptoms instead of global ED severity interacting with sleep and mood. Overall, this study represents an initial step in the investigation of EDs and sleep and mood dysregulation among an under-represented sample.

## 1. Introduction

Eating disorders (EDs) are severe psychological conditions that negatively impact individuals’ eating behaviours and perception of their bodies [1]. At their extreme, they can cause permanent health issues and may be lethal [2]. According to the *5th edition of the Diagnostic and Statistical Manual*, anorexia nervosa (AN) is a type of ED characterised by a restriction of energy intake, as well as abnormal body image and a fear of gaining weight. AN is the most-common of all EDs, reaching a prevalence of 2% to 4% in females [3] and 0.1% to 0.3% in males [4], although ED incidences appear to be increasing particularly among the younger demographic [3].

In addition to the detrimental effect of EDs on physical health, studies have shown that disordered eating behaviours and sleep problems are related [5], affecting over half of all individuals with EDs [6]. For example, Aspen and colleagues [7] found that up to 30% of females diagnosed with EDs also exhibited symptoms of clinical insomnia. In another study by Abdou and colleagues [8], through a comparison between females with AN or bulimia nervosa (BN) with healthy controls, it was found that those with EDs tended to report more symptoms of poor sleep, including insomnia, interrupted sleep, excessive daytime sleepiness, and parasomnias. Sleep quality also appears to be compromised, with patients with EDs reporting significantly lower sleep efficiency and longer sleep onset latency [6,8]. Larger-scale studies have shown similar patterns of findings. In a survey study involving 3790 women, Trace and colleagues [9] found that binge eating was related to poor sleep quality and insufficient sleep. Another large-scale prospective adolescent study with 12,082 participants found that disordered eating behaviours such as restrictive eating, compensation behaviours, and binge eating are all significantly associated with trouble initiating or maintaining sleep at seven-year follow-up [10]. In the context of AN specifically, sleep fragmentation appears to be a common experience in AN [11]. A literature review by Padez-Vieira and Afonso [12] summarises the existing evidence, finding poorer sleep quality and sleep efficiency, longer sleep onset latencies and more night awakenings, as well as early waking among AN patients, resulting in shorter amounts of sleep [13,14].

However, the correlation between ED and sleep issues is bi-directional, where sleep disturbances have also been found to affect eating behaviours [15]. For example, Kim and colleagues [6] demonstrated that individuals experiencing sleep problems are more likely to engage in binge eating and compensatory behaviours, while a lack of sleep in men is related to increased desire for high-calorie food [16].

Due to the close relationship between sleep and eating behaviours, it has been posited that the relationship is due to shared factors that have the ability to impact both aspects of life [7], such as mood dysregulation or stressful life events. In fact, after controlling for age and Body Mass Index (BMI), depression was able to fully mediate the relationship between binge eating disorder and insomnia [17]. Relatedly, mood disorders are often co-morbid with EDs: up to 80% of patients diagnosed with AN and BN also have a diagnosed mood disorder (with depression being the most common [18]) and are also commonly related to problems with sleep onset latency and insomnia [8]. In another empirical study by Evans and colleagues [19], it was also found that acute negative affect among individuals with EDs is linked to poor sleep quality.

### The Present Study

Due to its disproportionate prevalence among women than men, EDs have been traditionally treated as a “female” disease [20]. However, according to a study by Mayo and George [21] among male university students, a whopping 28% of men could qualify as having the risk of developing an ED, with increasing rates of prevalence highlighting the changing understanding of male EDs [22]. Studies that involve male participants in the investigation of sleep and EDs likewise show a significant relationship between sleep problems such as poor sleep quality and higher daytime sleepiness with ED diagnosis [13,23]. Along the same thread, the bi-directionality of this relationship was also shown in a large-scale study of 12,087 high school students, where dieting, fasting, and purging behaviours were significantly related to short sleepers among males [24]. A comparison between sexes illustrated that there may be different mechanisms at work in EDs between males and females, where social pressure to eat was significantly associated with insomnia and sleep problems among males, but not among females [25]. Therefore, the present study aimed to investigate the ED and sleep relationships specifically among males, in order to address this research gap.

To do so, the research aimed to make use of objective actigraphy data, which measure individuals’ activity over their sleep/wake cycles, together with validated questionnaires measuring ED severity and mood, to investigate the relationships between these constructs among males with AN. Overall, the aims of this research were to: (1) provide a general descriptive outline of sleep quality in male AN patients; (2) uncover the relationships between ED severity, sleep, and mood. Based on existing literature, we hypothesised that there are significant relationships between EDs and mood, as well as mood and sleep, where negative mood predicts both greater ED severity and poorer sleep.

## 2. Materials and Methods

The present study adopted a quantitative within-subject design and was approved by the University College London Institute of Education Research Ethics Committee (Ref. Z6364106/2018/07/44).

### 2.1. Participants

Participants were 33 male patients aged between 19 and 28 years of age (mean age = 23.38, SD = 2.61) who were diagnosed with AN. Recruitment was conducted via a convenience sample from various social media platforms, as well as advertisements in hospitals and universities in the London area. The demographic information of the participants are summarised in Table 1.

### 2.2. Materials

#### 2.2.1. Eating Disorder Examination Questionnaire

The assessment of ED severity was conducted using the Eating Disorder Examination Questionnaire (EDE-Q, ver. 17.0) [26], which consists of 28 items. Responses fall on a 6-point Likert scale, where higher EDE-Q scores correspond to more severe ED symptoms. The questionnaire shows robust psychometric properties with acceptable internal consistency (Cronbach’s alpha = 0.70 to 0.93) and test–retest reliability [27].

#### 2.2.2. Depression Anxiety Stress Scale

The assessment of mood was conducted using the Depression Anxiety Stress Scale (DASS) [28]. The questionnaire is composed of 3 subscales of 7 items each, assessing the severity of depression, anxiety, and stress symptoms, respectively, over the past week. Likewise, the DASS shows a good psychometric structure with the three subscales based on a confirmatory factor analysis [29], as well as acceptable internal consistency (Cronbach’s alpha = 0.87 to 0.94).

#### 2.2.3. Actigraphy Device

Actigraphy is a commonly used method of measuring activity during sleep/wake cycles, based on the notion that movement patterns can predict whether an individual is asleep or awake. Actigraphy devices perform measurements using an accelerometer embedded within a watch-like strap on the non-dominant wrist. For the purposes of the current study, the assessment of sleep behaviour was captured using the waterproof MotionWatch 8 by CamNtech. Sensitivity was set at 30-s intervals.

Actigraphy was chosen as an objective measure of sleep behaviour because of its non-invasive nature, which allows participants to behave in more naturalistic settings by sleeping in their homes rather than in a laboratory. During the time in which the data were collected, as it was during the height of the COVID-19 pandemic, actigraphy was also useful to minimise health risks.

The following data were derived from actigraphy measurements:

- Total sleep time: total time asleep in the night-time;
- Sleep efficiency: percentage of total time asleep out of the total time spent in bed;
- Sleep onset latency: time taken to fall asleep;
- Mobile time: percentage of time spent moving during a period of sleep;
- Fragmentation index: degree of movement during the night and an indication of sleep quality;
- Wake bout frequency: number of contiguous wake sections in the epoch-by-epoch wake/sleep categorisation;
- Wake bout duration: average length of each wake bout;
- Nap duration: total time asleep in the daytime;
- Nap frequency: number of times fallen asleep in the daytime.

All night-time and daytime actigraphy variables were averaged on a per-night basis. Data were first stored on the MotionWatch 8 during the period of data collection, then extracted using the Motionware software developed by the same manufacturer, CamNtech, when the device was retrieved from the participants.

### 2.3. Procedure

Informed consent was sought from the participants prior to joining the study. The actigraphy device was then mailed to the participant’s home address. The participants would be instructed to wear the device before bedtime for seven continuous days (i.e., a week) until the morning of the eighth day. Then, they would be asked to return the device by post.

Next, participants would receive the online EDE-Q and DASS questionnaires from the researchers and be asked to complete them on their own devices (e.g., smart phone, tablets, laptops). Due to the potentially triggering nature of the questions, participants were reminded of their rights to withdraw from the study. The completed questionnaires were sent back to the researchers via email.

### 2.4. Analytic Plan

All data were anonymised. Actigraphy data were converted into numerical values. All subsequent analyses were performed in IBM SPSS 27 for windows. Correlation analyses between variables were conducted using a two-tailed Pearson’s correlation.

## 3. Results

### 3.1. Descriptive Data

Table 2 describes the average sleep patterns of the participants.

The descriptive actigraphy data showed that, on average, the sleep duration of males with AN was 6.45 h/night (i.e., approximately 6 h 27 min; SD = 1.13), with a sleep efficiency of 74.98% (SD = 13.66). The participants typically fell asleep 0.52 h (i.e., approximately 31 min) after going to bed (SD = 0.60). There was 11.72% mobile time and an average fragmentation index of 39.64 (SD = 12.97), indicating relatively high activity levels during sleep. Average wake bouts of 37.37 (SD = 14.27) with a mean duration of 2.18 min (SD = 0.6) corroborated the above, indicating less continuous sleep.

However, daytime napping showed unusual patterns among the participants, with an average duration of 49.63 min (SD = 20.86) and a frequency of 4.68 naps/day (SD = 1.94). In reviewing sleep data over the course of 24 h, a visual inspection suggested that participants may be “chunking” their sleep into shorter bursts throughout the day at irregular hours. However, this unusual pattern needs further confirmation via the use of larger sample sizes.

Based on their relative standard deviations, it was noticeable that the participants varied primarily with regard to their sleep efficiency, fragmentation index, wake bouts, and nap durations.

In terms of questionnaire data, participants scored a mean of 4.77 (SD = 0.23) on the EDE-Q and 43.06 (SD = 2.57) on the DASS, with the various mean DASS subscale scores being 10.61 (Depression; SD = 2.47), 14.06 (Anxiety; SD = 2.57), and 18.39 (Stress; SD = 2.26).

### 3.2. Correlation Analysis

Two-tailed Pearson’s correlation was conducted between EDE-Q scores, including BMI, and actigraphy variables measuring sleep quality, namely total sleep time, sleep efficiency, mobile time, fragmentation index, nap duration, and nap frequency. Wake bout frequencies and duration were not considered as they represent similar constructs as mobile time.

Interestingly, correlation analysis (Table 3) demonstrated a significant positive relationship between sleep efficiency and BMI and between fragmentation index and mobile time. However, there were no significant relationships between ED severity and sleep measures.

To assess the relationship between ED severity and mood, a two-tailed Pearson’s correlation analysis was used (Table 4).

From the above findings, ED severity was significantly related to depression (*r*(33) = 0.40, *p* = 0.02). On the contrary, the relationship between EDE-Q and the DASS Anxiety subscale was not significant (*r*(33) = 0.04, *p* = 0.82). Neither was it significant with the DASS Stress subscale (*r*(33) = −0.30, *p* = 0.09) and global DASS scores (*r*(33) = 0.11, *p* = 0.55).

To investigate the relationship between mood and sleep quality in males with AN, a two-tailed Pearson’s correlation analysis showed that only the DASS Stress subscale was positively related to total sleep time (*r*(4) = 0.99, *p* = 0.01; Table 5).

To summarise, the study only found a significantly positive relationship between the EDE-Q score and the DASS Depression score and a significantly positive relationship between the DASS Stress score and total sleep time.

## 4. Discussion

The current study set out with two goals: (1) to provide a descriptive summary of sleep quality among males with AN and (2) to uncover the relationships between ED severity, sleep, and mood.

As the descriptive results showed, similar to their female counterparts, male participants with AN also experience decreased sleep quality. When comparing the obtained data with the average data derived from secondary sources, males with AN tended to sleep less, averaging 6.45 h/night as compared to 7.1/night in the general population based on a large-scale study involving 1.1 million participants in the United Kingdom, the United States, and the Netherlands [30]. Males with AN also tended to show poorer discrete sleep behaviours, including increased sleep latency (mean = 0.52 h compared to typical mean = 0.17 h; [31]) and fragmentation index (mean = 39.64 compared to typical mean = 6.1; [32]), as well as decreased sleep efficiency (mean = 74.98% compared to typical mean = 89%; [30]).

Based on the fragmentation index and mobile time findings, it appeared that males with AN had increased activity levels during sleep, corroborating the past findings by Lauer and Krieg [11], who theorised that the increased fragmented sleep among individuals with EDs is due to orexin and leptin dysregulation [7,15,33]. Orexin, a neuropeptide released by the hypothalamus, is implicated in both appetite regulation and wakefulness [33], where high levels are associated with hunger and increased wakefulness. Leptin, a hormone produced by adipose cells, is a marker of satiation and promotes sleepiness [11]. Therefore, dysregulation of these molecules in EDs would implicate not only feeding patterns, but also sleep behaviours. In fact, Sauchelli and colleagues [34] measured orexin-A concentrations in females with AN, as well as healthy controls and correlated these measurements with self-reported sleep quality. From that empirical study, it was found that poor sleep efficiency, sleep disturbances, and overall sleep quality were correlated with increased orexin concentration. Interestingly, although most literature regarding orexin tends to focus on its regulatory roles in feeding and sleep behaviours, recent studies have surfaced that implicate the orexin system in mood and cognition as well (see Chieffi et al. [35] for a review). Animal studies on mouse models without orexin-1 receptors found that these mice display altered behaviours related to depression and increased behaviours related to anxiety [36]. Additionally, complementing the findings of Sauchelli and colleagues [34], it was also found in a recent study that increased orexin plasma is found in patients with mood disorders such as depression and bipolar disorders as compared to control participants, as well as correlated with increased suicidal ideation [37], suggesting a multi-systemic influence of orexin on human function.

The findings of poor sleep efficiency among males with AN were corroborated by past studies such as Wheaton and colleagues [24], Delvenne and colleagues [38] and Della Marca and colleagues [39]. The underlying cause of this relationship is postulated to be due to poor nutrition as a result of food restriction in AN [11,33,40]. In the same study concerning sleep and EDs by Allison and colleagues [33], it was found that only individuals with AN suffered impaired sleep, whereas individuals with BN showed no statistical difference from healthy controls. According to the authors, starvation in AN is more prevalent and, therefore, could have contributed to poor sleep, similar to how food restriction and starvation among the general population are also related to sleep difficulties [11]. Additionally, an old study by Crisp and colleagues [40] supported the notion of poor nutrition being a potential factor contributing to poor sleep efficiency as it was found that patients with AN reported longer sleep duration upon undergoing refeeding treatment.

Relatedly, in addition to poor sleep, males with AN also experience longer sleep onset latency, indicative of insomnia, relative to the general population. This finding was supported by Levy and colleagues [14] and Lacey and colleagues [41]. Levy and colleagues [14] suggested that insomnia may be attributable to a low BMI, which was confirmed by the findings of Lacey and colleagues [41] comparing the sleep quality of patients with AN before and after weight gain. Upon gaining weight, Lacey and colleagues [41] found that individuals with AN experienced improvements in overall sleep quality, as well as less severe insomnia. However, it should be noted that there are contrary findings relating BN and binge eating disorder more strongly to insomnia than AN [17,25] and that insomnia was instead positively correlated with the BMI [23]. A potential factor that may account for these contrasting findings lies in binge eating behaviour. In a related study by Yeh and Brown [42], it was found that binge eating behaviour mediated the relationship between a high BMI and insomnia. In the context of AN, it may explain why individuals with AN may suffer insomnia even while reporting a low BMI due to the potential presence of binge eating AN subtypes within the sample. Unfortunately, this finding was later overturned by Kenny and colleagues [17], where binge eating frequency no longer predicted insomnia severity after controlling for participant age, BMI, depression, and anxiety. Due to the contrasting findings, the relationship between insomnia/longer sleep onset latency and the various EDs can be, therefore, clarified with a future comparative study with all categories of EDs represented in the participant sample.

With regard to the second research question, based on the correlation findings, this study did not find any direct relationships between ED severity and sleep behaviour. Therefore, we further tested the relationship between ED and mood, as well as mood and sleep. While there was a significant correlation between ED severity and depression, depression itself was unrelated to other sleep measures. On the other hand, anxiety was positively correlated with total sleep time, but unrelated to ED severity. There is, therefore, a lack of evidence from this study that corroborates the literature outlined above. There has been some evidence illustrating that sleep difficulties experienced in EDs may be qualitatively different from sleep difficulties associated with mood dysregulation [14]. In fact, comparing AN with depression, Delvenne and colleagues [38] found that patients with AN showed even poorer sleep outcomes in terms of more frequent and prolonged sleep fragmentation. Overall, it appears that further research is needed to tease apart the nature of sleep difficulties in EDs, as well as the potential mediator of mood dysregulation.

### Limitations and Future Studies

There are several limitations of the study to be acknowledged. Firstly, actigraphy has limited validation for daytime variables such as nap frequency and duration [43], as data may be altered due to other movements such as breathing, generic arm movement, and environmental factors. Therefore, data from actigraphy may be bolstered with the use of other measures (e.g., sleep diaries) that can corroborate the data obtained. In the present study specifically, there were also missing data stemming from participants’ unwillingness to wear or forgetfulness in wearing the actigraphy device for the full duration of a week.

Secondly, due to practical constraints, healthy controls were not recruited for this study. Therefore, the present findings could only be compared with secondary data available from other published studies. However, as there appears to be a general worsening of sleep quality over time [44], it is difficult to establish if the sleep difficulties experienced by participants with EDs in this study are attributable to the EDs or to the larger societal trend. Including a control sample would be helpful in determining if these sleep problems are significantly more severe among individuals with EDs as compared to those without.

Thirdly, self-reported questionnaires used as a measure of ED severity and mood may be subject to social desirability biases or recall biases. The severity of ED and mood dysregulation may be more objectively assessed by a professional clinician.

Overall, based on the limitations of the current study, further research using other objective sleep measurements is needed. Future studies may also consider incorporating a control group. Similar study designs may also be extended to males with other EDs such as BN or binge eating disorders. Based on the limited findings of the present study, another potential study extension may lie in investigating specific disordered eating behaviours and their relationships with mood and sleep, instead of global ED severity. In summary, further research with larger sample sizes using a multi-disciplinary approach by combining objective sleep measures, environmental factor data, and hormonal measures would provide a firmer approach towards the causal pathways of sleep disturbances.

## 5. Conclusions

To conclude, this study aimed to find a relationship between eating disorder, mood, and sleep. From descriptive actigraphy data, this research found that, just like their female AN counterparts, male AN patients also suffer from poor sleep quality, characterised by insomnia, fragmented sleep, poor sleep efficiency, and poor total sleep time. However, based on the correlational analyses, the study failed to find sufficiently significant relationships between ED severity, sleep, and mood, except for a significant positive relationship between the EDE-Q and DASS Depression scores, as well as another significant positive relationship between the DASS Stress score and total sleep time. Nonetheless, the present study represents an initial step in the investigation of EDs among males, as well as the relationship between EDs, mood, and sleep in a typically under-represented population.

## Figures and Tables

**Table 1:** Demographic details of participants.

Variable	Number	Mean	Standard Deviation	Percentage (%)
Age	33	23.38	2.61	-
BMI (kg/m^2^)	33	19.23	1.04	-
**Education**				
Up to high school	4	-	-	12.1
Bachelor’s degree	21	-	-	63.6
Post-graduate degree	8	-	-	24.2
**Employment Status**				
Employed	3	-	-	9.1
Unemployed	30	-	-	90.9
**Marital Status**				
Single	3	-	-	9.1
Married	30	-	-	90.9
**Children**				
None	31	-	-	93.9
One	1	-	-	3
Two	1	-	-	3

Bolded words refer to variable categories.

**Table 2:** Descriptive actigraphy data.

Actigraphy Variable	Mean	Standard Deviation
Total sleep time (h)	6.45	1.13
Sleep efficiency (%)	74.98	13.66
Sleep onset latency (h)	0.52	0.60
Mobile time (%)	11.72	6.07
Fragmentation index	39.64	12.97
Wake bouts	37.37	14.37
Wake bout duration (min)	2.18	0.60
Nap duration (min)	49.63	20.86
Nap frequency	4.68	1.94

**Table 3:** Correlation probabilities between ED and sleep.

	1	2	3	4	5	6	7
1. EDE-Q							
2. BMI	0.48						
3. Total sleep time	0.28	0.91					
4. Sleep efficiency	0.21	0.02 *	0.83 ^a^				
5. Mobile time	0.21	0.14 ^a^	0.70	0.56 ^a^			
6. Fragmentation index	0.18 ^a^	0.18 ^a^	0.62	0.08 ^a^	0.00 **		
7. Nap duration	0.65	0.89 ^a^	0.35 ^a^	0.81 ^a^	0.70	0.76	
8. Nap frequency	0.55	0.89	0.42 ^a^	1	0.84	0.87	0.07

** *p* < 0.01; * *p* < 0.05; ^a^ negative correlation coefficient.

**Table 4:** Correlation probabilities between ED and mood.

	1	2	3	4
1. EDE-Q				
2. DASS (Depression)	0.02 *			
3. DASS (Anxiety)	0.82	0.77		
4. DASS (Stress)	0.09 ^a^	0.03 *^a^	0.83	
5. DASS	0.55	0.00 **	0.00 **	0.03 *

** *p* < 0.01; * *p* < 0.05; ^a^ negative correlation coefficient.

**Table 5:** Correlation probabilities between mood and sleep.

	1	2	3	4	5	6	7	8	9
1. DASS (Depression)									
2. DASS (Anxiety)	0.77								
3. DASS (Stress)	0.03 *^a^	0.83							
4. DASS	0.00 **	0.00 **	0.03 *						
5. Total sleep time	0.59 ^a^	0.47	0.01 **	0.17					
6. Sleep efficiency	0.30 ^a^	0.38	0.76 ^a^	0.99 ^a^	0.83 ^a^				
7. Mobile time	0.27	0.63 ^a^	0.61	0.72	0.70	0.56 ^a^			
8. Fragmentation index	0.33	0.71 ^a^	0.53	0.63	0.62	0.08 ^a^	0.00 **		
9. Nap duration	0.17	0.74 ^a^	0.45 ^a^	0.80 ^a^	0.35 ^a^	0.81 ^a^	0.70	0.76	
10. Nap frequency	0.31	0.99 ^a^	0.53 ^a^	0.95 ^a^	0.42 ^a^	1	0.84	0.87	0.07

** *p* < 0.01; * *p* < 0.05; ^a^ negative correlation coefficient.

## Data Availability

Full data obtained may be made available upon request to the corresponding author.

## Abbreviations

The following abbreviations are used in this manuscript:

ED	Eating disorder
AN	Anorexia nervosa
EDE-Q	Eating Disorder Examination Questionnaire
DASS	Depression Anxiety Stress Scale
BN	Bulimia nervosa
BMI	Body Mass Index
